# Mechanism of selective anticancer activity of isothiocyanates relies on differences in DNA damage repair between cancer and healthy cells

**DOI:** 10.1007/s00394-019-01995-6

**Published:** 2019-05-23

**Authors:** Aleksandra Hać, Joanna Brokowska, Estera Rintz, Michał Bartkowski, Grzegorz Węgrzyn, Anna Herman-Antosiewicz

**Affiliations:** 1 Department of Medical Biology and Genetics, Faculty of Biology, University of Gdańsk, Wita Stwosza 59, 80-308 Gdańsk, Poland; 2 Present Address: Department of Molecular Biology, Faculty of Biology, University of Gdańsk, Wita Stwosza 59, 80-308 Gdańsk, Poland

**Keywords:** DNA replication, Genotoxic stress, Phenethyl isothiocyanate, Sulforaphane, Prostate cancer

## Abstract

**Purpose:**

Isothiocyanates (ITCs) are compounds derived from *Brassica* plants with documented anticancer activity. Molecular mechanisms of their selective activity against cancer cells are still underexplored. In this work, the impact of ITC on DNA replication and damage was compared between PC-3 prostate cancer cells and HDFa normal fibroblasts as well as PNT2 prostate epithelial cells.

**Methods:**

Cells were treated with sulforaphane or phenethyl isothiocyanate. [^3^H]thymidine incorporation and the level of histone γH2A.X were estimated as indicators of DNA replication and double-strand breaks (DSB), respectively. Levels of HDAC3, CtIP, and p-RPA were investigated by immunoblotting. Comet assay was performed to visualize DNA damage.

**Results:**

ITCs inhibited DNA replication in all tested cell lines, and this activity was independent of reactive oxygen species of mitochondrial origin. It was followed by DSB which were more pronounced in cancer than noncancerous cells. This difference was independent of HDAC activity which was decreased in both cell lines when treated with ITCs. On the other hand, it correlated with faster removal of DSB, and thus, transient activation of repair proteins in normal cells, while in PC-3 prostate cancer, cell DNA repair was significantly less effective.

**Conclusion:**

DNA damage induced by ITCs is a consequence of the block in DNA replication which is observed in both, cancer and normal cells. Selective antiproliferative activity of ITCs towards cancer cells results from less efficient DNA repair in cancer cells relative to normal cells.

## Introduction

Vegetables from Brassicaceae family, such as broccoli, radish, cauliflower, or cabbage, are a rich source of glucosinolates [1]. They are enzymatically hydrolyzed by a plant myrosinase to isothiocyanates, nitriles, thiocyanates, and other products [2]. Isothiocyanates (ITCs) have been intensively studied in recent years, mainly because of their chemopreventive and anticancer activities. It has been shown that they disturb multiple steps of the carcinogenesis and ultimately inhibit tumor growth in such organs as stomach, liver, colon, esophagus, lung, bladder, prostate, or breast (for review, see [3, 4]). Among them, the most commonly studied are 1-isothiocyanato-4-methylsulfinylbutane (sulforaphane, SFN) and phenethyl isothiocyanate (PEITC). Both in vitro and in vivo studies have shown that chemopreventive and anticancer activities of isothiocyanates are associated with modulation of phase I and II biotransformation enzyme activities, induction of the cell-cycle arrest and/or apoptosis of the cancer cells as well as inhibition of angiogenesis and metastasis [4]. Interestingly, numerous publications indicate that ITCs are much more potent towards cancer than noncancerous cells. For instance, allyl isothiocyanate (AITC) and phenyl isothiocyanate (PITC) inhibited growth of A549 and H1299 lung cancer cells but not HBEC normal human bronchial epithelial cells [5]. Nontumorigenic human mammary epithelial MCF-10A cells were resistant to SFN-induced oxidative stress and cell death [6]. Similarly, sulforaphene and erucin were much less potent towards MCF10A than a panel of breast cancer cell lines [7, 8].

One of the mechanisms underlying ITCs’ cytotoxic activity is their genotoxicity. DNA damage checkpoint with the activation of ATM and Chk2 kinases, phosphorylation, and nuclear foci formation by the phosphorylated histone H2A.X (γH2A.X) and subsequent G2/M cell-cycle arrest has been initially documented in SFN-treated prostate cancer cells [9]. Double-strand breaks (DSB) and homologous recombination repair induction have been observed in HeLa cells treated with SFN (10–50 µM) [10]. PEITC induced DNA damage in HCT116 and HT29 colon cancer cells [11].

Different mechanisms of ITCs genotoxicity have been proposed. It has been shown that ITCs induce oxidative stress which is responsible for induction of DNA damage checkpoint [12, 13]. Moreover, transient DNA oxidative modification (8-oxo-dG) has been reported in lung cancer cells treated with SFN for 0.5 h [14]. On the other hand, DNA damage might also result from perturbations in DNA metabolism. It has been shown that ITCs might modify cysteine residues in topoisomerase IIα which leads to TopIIα-DNA covalent adducts and cells death [15]. This enzyme is highly upregulated in cancer cells; thus, its inactivation by ITCs may partially explain their selective activity towards transformed cells. However, exact dependencies between DNA replication and damage in cancer and noncancerous cells treated with ITCs have not been investigated.

The aim of this work was to elucidate whether ITC-induced DNA damage is associated with DNA replication block and whether it differs between PC-3 prostate cancer cells and normal cells: HDFa human dermal fibroblasts and PNT2 prostate epithelial cells.

## Materials and methods

### Reagents

SFN (purity ≥ 98%) and PEITC (purity ≥ 98%) were obtained from LKT Laboratories (St. Paul, MN). Culture media, fetal bovine serum (FBS), penicillin–streptomycin–neomycin antibiotic mixture, and trypsin were purchased from Thermo Fisher Scientific (Waltham, MA). Muse kits were purchased from Merck Millipore (Billerica, MA). Sulforhodamine B (SRB), anti-tubulin, anti-rabbit, anti-mouse, and anti-β-actin antibodies conjugated with horseradish peroxidase (HPR) were purchased from Sigma-Aldrich (St. Louis, MO). Antibodies against p-RPA (Ser4/Ser8; #A300-245A) and CtIP (#A300-488A) were from Bethyl Laboratiories (Montgomery, TX) and against HDAC3 (#sc-376957) from Santa Cruz Biotechnology (Dallas, TX).

### Cell culture

Monolayer cultures of PC-3 cells were maintained in F12-K Nutrient Mixture medium supplemented with 9% (v/v) fetal bovine serum (FBS) and penicillin–streptomycin mixture. Rho0 derivatives of PC-3 cells, which are depleted of mitochondrial DNA, were obtained, as described in [16]. They were maintained as PC-3 cells, but medium was additionally supplemented with 100 ng/ml ethidium bromide, 4.5 mg/ml glucose, 100 μg/ml sodium pyruvate, and 50 μg/ml uridine to compensate for the respiratory metabolism deficit. HDFa and PNT2 cells were maintained in DMEM or RPMI1640, respectively, supplemented with 10% (v/v) FBS and antibiotics. Each cell line was maintained at 37 °C in a humidified atmosphere with 5% CO_2_.

### Cell-viability assay

Cells were seeded in 96-well plate. After 24 h, cells were incubated in medium supplemented with ITCs at different concentrations. Controls were treated with DMSO. Viability of cells was assessed using SRB assay. After 24 h medium was removed and 100 μl of 20% (w/v) aqueous solution of ice-cold trichloroacetic acid (TCA) was added to wells for 1 h. Plates were washed with water, allowed to air dry, and stained with 100 μl of 0.4% SRB solution in 1% acetic acid for 15 min. Cells were extensively washed 5 times with 1% acetic acid and dried. After the addition of 10 mM Tris base (pH 10.5, 150 μl/well), absorbance was measured at 570 nm with a reference filter of 660 nm in Victor3 microplate reader.

### DNA synthesis

Cells were seeded in 12-well plate and allowed to attach overnight. The next day, cells were simultaneously treated with 2 µCi/ml of methyl-^3^H-thymidine and either indicated concentration of ITCs or vehicle (DMSO; control) for 3 h, or with 40 µM SFN, 10 µM PEITC or DMSO (control) for indicated time. When incubations finished, cells were fixed with 5% (w/v) aqueous solution of TCA for 1 h and washed with another portion of TCA for 30 min. The acid-insoluble material was dissolved in 0.1 mol/L KOH overnight at 4 °C and aliquots were used to determine the radioactivity using liquid scintillation counter (MicroBeta^2^ Microplate Counter, Perkin Elmer).

### Cell cycle and γH2A.X amount analysis

Cells were seeded on 60 mm dishes and incubated overnight. The next day, cells were treated with 40 μM SFN, 10 μM PEITC or 20 μM etoposide for desired time. Both floating and attached cells were collected and washed with ice-cold PBS, proceeded with the Muse^TM^ Cell-Cycle Kit or Muse^TM^ H2A.X Activation Dual Detection Kit (Merck Millipore, Germany) according to manufacturer’s instruction, and analyzed using Muse™ Cell Analyzer flow cytometry. 5000 counts were measured per sample.

### Comet assay

Cells were seeded on 6-well plate and allowed to attach overnight. The next day, cells were treated with SFN (40 µM), PEITC (10 µM), vehicle (DMSO, control), or 20 µM etoposide for 3 h and collected by trypsinization in a dim light. After washing with ice-cold PBS (Ca^2+^ and Mg^2+^ free), cells were combined with a low melting agarose in PBS of 37 °C and placed on agarose-coated slides. DNA fragmentation was measured by the alkaline comet assay using CometAssay^®^ (Trevigen) according to manufacturer’s instructions. Slides were visualized under fluorescent microscope (DMI4000B, Leica). Experiment was performed in at least two independent replicates. Analysis of comets was performed using ImageJ with Open Comet plug.

### HDAC I/II activity measurement

Cells were seeded in 96-well white clear bottom plate in duplicate and incubated overnight. The next day, cells were treated with SFN (20 μM or 40 μM), PEITC (5 μM or 10 μM), vehicle (DMSO, control), or 200 nM trichostatin A (TSA) as a positive control for 3 h. The activity of histone deacetylases (I/II) was examined using HDAC-Glo™ I/II Assay (Promega) according to the manufacturer’s instructions. The activity of controls was taken as 100%. Experiment was performed in two independent replicates.

### Western blot analysis

6 × 10^5^ cells were seeded on 100 mm dishes and grown overnight. The next day, cells were treated with ITCs or vehicle (DMSO, control) for 3 h and either collected or washed and cultured in a drug-free media for additional 3 or 16 h. After harvesting, cells were lysed for 30 min on ice with the lysis solution (50 mM Tris (pH 7.5), 1% Triton X-100, 150 mM NaCl, 0.5 mM EDTA, protease, and phosphatase inhibitor cocktails (Roche Diagnostics, Germany) and centrifuged (15,500*g*, 4 °C, 20 min). Immunoblots were performed as previously described [17]. Briefly, proteins were resolved by SDS–polyacrylamide gel electrophoresis (SDS-PAGE) and transferred onto PVDF membrane. The membrane was blocked with PBS buffer containing 0.1% Tween-20 and 5% non-fat dry milk and incubated with the desired primary antibody overnight at 4 °C. The membrane was treated with appropriate secondary antibody for 1 h at room temperature. The immunoreactive bands were visualized by enhanced chemiluminescence reagent (Thermo Scientific Pierce, Rockford, IL). The blots were stripped and reprobed with anti-β-actin antibody to normalize for differences in protein loading. The immunoblotting for each protein was performed at least three times using independently prepared lysates.

### Statistical analysis

Data were analyzed using the GraphPad Prism software. *T* test or one-way ANOVA, followed by Bonferroni’s multiple comparison test, was used to determine statistical significance of the differences in the measured variables between the tested groups. Difference was considered significant at *p *< 0.05.

## Results

### Sulforaphane and PEITC inhibit DNA replication in both cancer and noncancerous cells

**Fig. 1:**
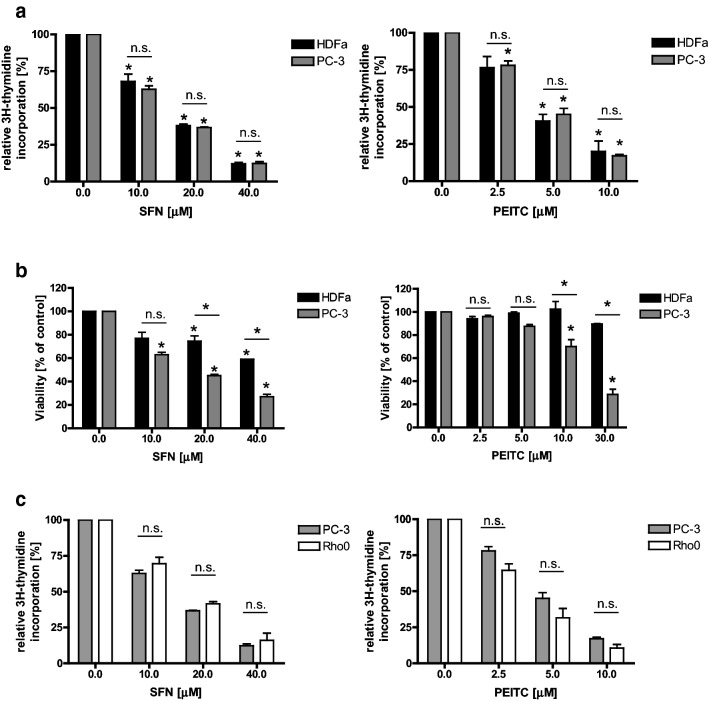
ITCs inhibit replication in both cancer and normal cells to a similar extend and in ROS-independent manner, although cancer cells viability is lower than normal cells. Prostate cancer cells (PC-3 and their Rho0 derivatives) and normal dermal fibroblasts (HDFa) were treated for 3 h (**a**, **c**) or 24 h (**b**) with indicated concentrations of SFN, PEITC or vehicle (DMSO; 0). **a**, **c** Cells were cultured in the presence of 2 µCi/ml of methyl-^3^H-thymidine. Thymidine incorporation was determined by liquid scintillation. Radioactivity of controls was taken as 100%. The results are expressed as the mean ± SE of 3 independent experiments. **b** Cell viability was assessed by SRB method. Each point is mean ± SE of two experiments done in triplicate. Significantly different at *p *< 0.01 (*) compared with control or between cell lines by one-way ANOVA followed by Bonferroni’s multiple comparison test; *n.s.* non significant

To elucidate whether SFN or PEITC influence DNA replication in cancer and noncancerous cells, we analyzed the level of [^3^H]thymidine incorporation into DNA of PC-3 prostate cancer cells and HDFa fibroblasts. Cells were grown in control conditions or exposed to different concentrations of tested ITCs for 3 h (too short time to observe apoptosis). As shown in Fig. 1a, SFN and PEITC in a dose-dependent manner reduced DNA synthesis in both cell lines. Interestingly, viability of tested cells after 24-h treatment differed: PC-3 cells were more sensitive to ITCs than healthy cells (Fig. 1b). It is worth noting that PEITC is more active than SFN and at lower concentrations reveals effects similar to SFN in terms of DNA replication inhibition; thus, for majority of subsequent experiments, we used 40 μM SFN and 10 μM PEITC.

### DNA replication inhibition is independent of ROS of mitochondrial origin

Anticancer activity of ITCs is connected with the elevation of oxidative stress which—at least partially—is due to inhibition of mitochondrial respiratory chain complexes [12, 13, 18]. To elucidate whether DNA replication inhibition is caused by reactive oxygen species (ROS) of mitochondrial origin, we compared [^3^H]thymidine incorporation in PC-3 cells and their Rho0 derivatives treated with SFN or PEITC. Rho0 cells do not contain mitochondrial DNA which codes for, inter alia, some components of mitochondrial respiratory chain complexes, thus are devoid of them. Cells were obtained and described by us previously [16]. As shown in Fig. 1c, ITCs blocked DNA replication to similar extent in both cell lines.

### ITCs induce DNA double-strand breaks more potently in cancer cells than in normal fibroblasts, and this process is preceded by DNA replication block

**Fig. 2:**
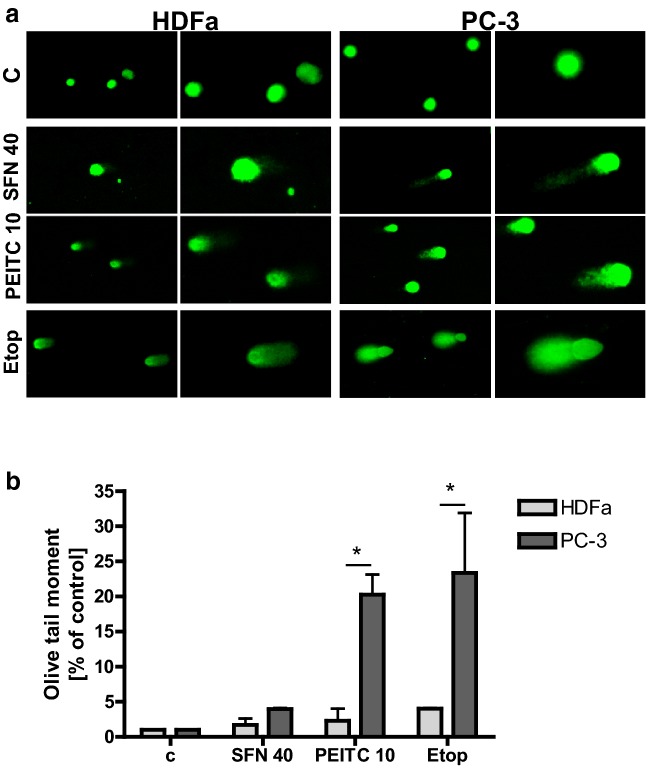
ITCs induce DNA damage. Prostate cancer cells (PC-3) and normal dermal fibroblasts (HDFa) were treated with DMSO (C), PEITC (10 µM), SFN (40 µM) or etoposide (20 µM) for 3 h. Alkaline comet assay was performed as described in “Materials and methods”. Experiment was performed in at least two independent replicates. **a** Representative images for each condition are shown. Magnifications of selected regions are shown on the right panels. **b** Olive tail moment was calculated to assess DNA integrity. Significantly different at *p *< 0.01 (*) between cell lines by one-way ANOVA followed by Bonferroni’s multiple comparison test

It has been reported previously that ITCs induce DNA damage [9–11, 15, 18, 19]. To compare its extent in cancer and noncancerous cells, we performed comet assay using PC-3 and HDFa cells treated with SFN, PEITC or topoisomerase inhibitor, etoposide, as a positive control. As expected, etoposide was the most powerful inducer of DNA damage which was evident as the largest comet tail (Fig. 2a). Olive tail moment, scored as general parameter of DNA integrity, was higher in cancer cells than noncancerous cells, although statistical significance was observed only for PEITC and etoposide treatment (Fig. 2b).

**Fig. 3:**
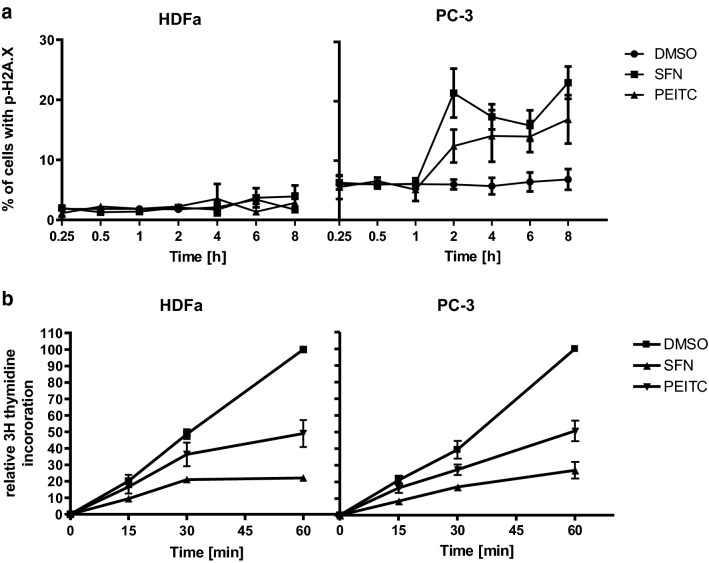
Inhibition of replication by ITCs is a rapid process, preceding genotoxic stress appearance, and occurs with the same kinetics in normal and cancer cells. **a** HDFa and PC-3 were treated with 40 µM SFN, 10 µM PEITC or equal amount of pure vehicle (DMSO) for indicated times and percentage of cells with p-H2A.X (γH2A.X) was determined by flow cytometry. **b**  HDFa and PC-3 cells were treated as in **a** in the presence of 2 µCi/ml of methyl-^3^H-thymidine. Thymidine incorporation was determined by liquid scintillation. Radioactivity in control samples at 60 min was taken as 100%. The results are expressed as the mean ± SE of 2–5 independent experiments

To analyze kinetics of DSB appearance in cancer and normal cells we performed cytometric evaluation of the amount of γH2A.X histone in different timepoints after treatment with ITCs. Significant elevation of the γH2A.X histone amount was detected after 2 h of treatment of PC-3 cells with 40 μM SFN or 10 μM PEITC (Fig. 3a). Importantly, the γH2A.X level in HDFa increased weakly or did not at all in cells treated with SFN or PEITC as compared to the level seen in untreated cells (Fig. 3a).

As it has been shown that ITC-induced DNA damage might be connected with the replication stress, we performed a time course evaluation of DNA replication in control cells and cells treated with ITCs. We observed that significant inhibition of DNA replication takes place already after 15–30 min of treatment with either SFN or PEITC in both HDFa and PC-3 cells (Fig. 3b) which indicates that DNA replication block precedes DNA damage.

### Cells treated with ITCs induce DNA damage response and S-phase cell-cycle arrest, which is stable in normal but not in cancer cells

We analyzed cell-cycle distribution of PC-3 and HDFa cells treated or not with ITCs for 6, 16, or 24 h. In normal cells, tested ITCs increased percentage of S-phase cells already after 6 h, but the most significant was at 16 h and was accompanied by decreased amounts of cells in G0/G1 phase (especially in case of PEITC-treated cells). After 24 h, cell-cycle profile in PEITC- or etoposide-treated HFDa cells resembled profile in DMSO-treated cells (Table 1). Interestingly, percentage of PC-3 cells in S phase increased moderately after 6 h of treatment with ITCs but later on reached control level and fraction of cells in G2/M phases increased at 16 h (although it did not reach statistical significances compared with controls). Topoisomerase inhibitor increased amount of cells in both, S and G2/M phases in PC-3 after 24-h treatment. It might indicate that S-phase checkpoint, which is connected with replication stress and DSB, is more efficient in normal than cancer cells.

**Table 1:** Isothiocyanates differently affect cell-cycle progression in fibroblasts and prostate cancer cells

Hours (h)	Cells	DMSO	SFN	PEITC	Etop
6	HDFa				
	G0/G1	83.0 ± 2.2	81.9 ± 1.9	81.3 ± 2.0	
	S	5.2 ± 1.0	8.1 ± 2.2	11.1 ± 2.9	
	G2/M	11.8 ± 1,3	10.0 ± 1.5	7.6 ± 2.1	
6	PC-3				
	G0/G1	53.5 ± 3.1	49.8 ± 3.7	48.4 ± 3.7	
	S	12.5 ± 0.6	16.2 ± 3.6	16.1 ± 3.5	
	G2/M	34.0 ± 3.6	34.0 ± 4.4	35.5 ± 4.3	
16	HDFa				
	G0/G1	83.0 ± 1.9	78.2 ± 0.7	75.2 ± 0.7	
	S	5.4 ± 1.4	10.6 ± 1.6	14.8 ± 2.1*	
	G2/M	11.6 ± 2.6	11.2 ± 2.1	10.0 ± 2.7	
16	PC-3				
	G0/G1	58.9 ± 6.9	51.0 ± 2.2	51.1 ± 2.8	
	S	11.5 ± 0.6	14.2 ± 1.1	11.0 ± 0.8	
	G2/M	29.6 ± 6.4	34.8 ± 2.5	37.9 ± 1.9	
24	HDFa				
	G0/G1	81.2 ± 2.0	84.2 ± 1.4	79.6 ± 2.8	81.6 ± 1.2
	S	7.8 ± 3.6	8.6 ± 2.6	8.9 ± 1.2	8.7 ± 1.9
	G2/M	11.0 ± 2.5	7.2 ± 1.9	11.5 ± 3.7	9.7 ± 1.1
24	PC-3				
	G0/G1	58.3 ± 4.0	53.1 ± 2.8	50.0 ± 3.1	27.1 ± 5.6***
	S	14.6 ± 2.9	14.4 ± 2.1	14.6 ± 2.1	28.5 ± 1.4**
	G2/M	27.1 ± 4.0	32.5 ± 5.0	35.4 ± 4.6	44.4 ± 4.2*

Human dermal fibroblasts (HDFa) and prostate cancer cells (PC-3) were treated for indicated time with DMSO (control), 40 µM SFN, 10 µM PEITC or 20 µM etoposide (Etop; positive control). The results are expressed as the mean ± SE of 3 independent experiments

Statistical significance of differences between means was tested by Bonferroni’s multiple comparison test (*p ≤ 0.05, **p ≤ 0.01, and ***p ≤ 0.001 between DMSO and SFN or PEITC)

### Lower level of DSB in normal cells does not result from lower proliferation rate or differences in HDAC activity

**Fig. 4:**
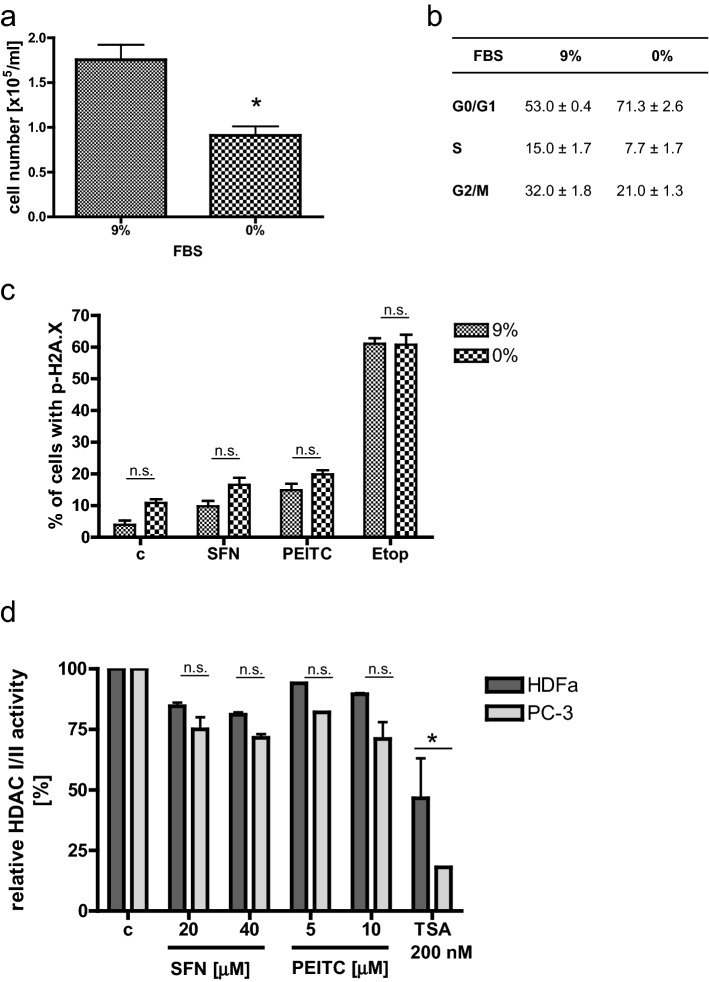
ITC-induced DNA damage is independent from cells proliferation rate and HDAC activity. PC-3 cells were cultured for 72 h in medium supplemented with 9% (control) or 0% fetal bovine serum (FBS) to decrease proliferation rate. Then cells were harvested to determine **a** cells number and **b** cell-cycle distribution, or **c** were treated with the vehicle (DMSO; c), 40 µM SFN, 10 µM PEITC or 20 µM Etoposide (Etop; positive control) for 3 h and the percentage of cells with p-H2A.X was determined by flow cytometry. The results are expressed as the mean ± SE of 3 independent experiments. **d** HDFa and PC-3 cells were treated for 3 h with indicated concentrations of SFN, PEITC, equal amount of the vehicle (DMSO; c) or 200 nM TSA (positive control). HDAC I/II activity was measured by HDAC I/II Glo Assay and activity of controls was taken as 100%. The results are expressed as the mean ± SE of two independent experiments. The statistical significance of differences between respective samples was determined by *t* test (**a**) or one-way ANOVA followed by Bonferroni’s multiple comparison test (**c**, **d**), where asterisk indicates significant differences between groups (*p *< 0.05); *n.s.* non significant

Cell-cycle analysis shows that more cancer than normal cells proliferates (S-phase fraction of PC-3 cells is about 13%, while HDFa—6%; Table 1). To elucidate if this feature is responsible for different degrees of DNA DSB, we slowed down proliferation of PC-3 prostate cancer cells and determined the level of γH2A.X histone in control and ITC-treated cells. As shown in Fig. 4a, b, 72-h serum deprivation led to a decrease in a number of PC-3 cells which resulted from G0/G1 cell-cycle arrest. Interestingly, the amount of DNA DSB assessed by the amount of γH2A.X histone was similar in cells characterized by their typical or slower proliferation rates and treated with SFN, PEITC or etoposide, used as a positive control (Fig. 4c).

It has been shown that ITCs inhibit histone deacetylases (HDAC) [20]. Acetylated DNA is more sensitive to DNA-damaging agents. Moreover, histone acetylation influences replication fork velocity, and thus, genome stability [21]. In addition, acetylation of some enzymes engaged in DNA repair regulates their stability [22]. Thus, we compared activity of HDAC in ITC-treated normal and cancer cells. Figure 4d shows that ITCs inhibited HDACI/II, indeed; however, a degree of this inhibition was similar in HDFa and PC-3 cells, while it was still lower than that in cells treated with TSA (HDAC I/II inhibitor; a positive control).

### DNA repair is more efficient in normal than cancer cells

**Fig. 5:**
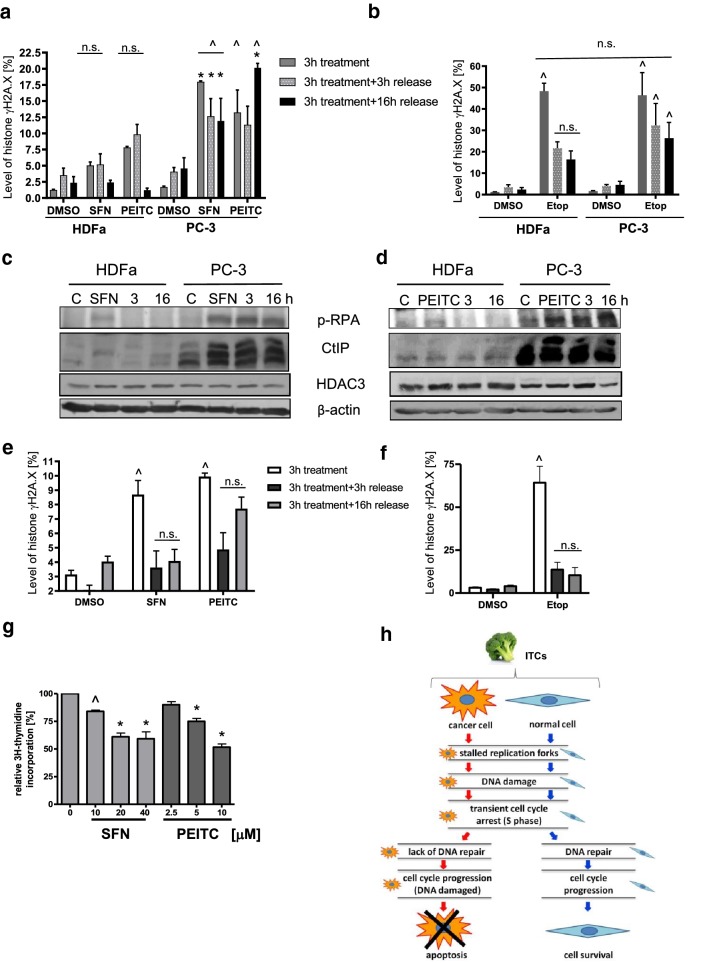
DNA repair is more efficient in normal than cancer cells. HDFa and PC-3 (**a**, **b**) or PNT2 (**e**, **f**) were treated with DMSO (control), 40 µM SFN, 10 µM PEITC or 20 µM Etoposide (Etop) for 3 h, then, either cells were collected or medium was exchanged for a drug-free one and cells were allowed to recover for additional 3 or 16 h. Percentages of cells in described above variants were determined by flow cytometry. The statistical significance of differences between respective samples was determined by one-way ANOVA followed by Bonferroni’s multiple comparison test, where ^ indicates significant difference between ITCs or etoposide-treated and respective control (DMSO-treated cells) and * indicates significant difference between cancer and noncancerous cells (*p *< 0.01); *n.s.* non significant. **c**, **d** Levels of p-RPA32 (Ser4/Ser8), CtIP and HDAC3 proteins were determined by immunoblotting. The blots were stripped and reprobed with anti-β-actin antibody to ensure equal protein loading. Shown are representative images of three independent experiments. **g** ITCs inhibit DNA replication in PNT-2 prostate epithelial cells. Cells were cultured in the presence of 2 µCi/ml of methyl-^3^H-thymidine. Thymidine incorporation was determined by liquid scintillation. Radioactivity of controls was taken as 100%. The results are expressed as the mean ± SE of two or three independent experiments. Significantly different at *p *< 0.001 (*) or *p* < 0.05 (^) compared with control by one-way ANOVA followed by Bonferroni’s multiple comparison test. **h** Summary of the present study: ITCs inhibit DNA replication in both, cancer and noncancerous cells which leads to DNA damage. DNA damage response is more efficient in normal than cancer cells, thus normal cells recover, while cancer cells continue cell cycle with unrepaired DNA which leads to their death

Elevated levels of DSB in cancer cells might result from inefficient DNA repair compared to normal cells. To validate such hypothesis, we treated both cell lines with SFN, PEITC or etoposide (as a positive control) for 3 h, replaced medium, and analyzed γH2A.X level after 3 or 16 h of culturing in a drug-free medium. As shown in Fig. 5a, b, γH2A.X was reduced much more efficiently in normal than cancer cells.

To investigate this phenomenon in more details, we analyzed the levels of two proteins engaged in DNA damage repair: p-RPA32 (Ser4/Ser8), necessary for each major DNA repair pathway, and CtIP which is a key player in homologous recombination repair activated by DSB. Figure 5c, d shows that upon ITCs treatment, the phosphorylation of RPA32 at Ser4/Ser8 increased in both cell lines; however, after removal of genotoxic agents, it decreased in HDFa, while remained at a high level in PC-3 cells. Similar trend was observed in the case of CtIP level. Stability of CtIP is inversely regulated by its acetylation. As it is a target of HDAC3, we determined whether ITCs influence HDAC3 level. As shown in Fig. 5c, d, HDAC3 level in ITC-treated HDFa cells increased and remained at this level in cells cultured without SFN or PEITC. In PC-3 cells, 3-h exposition to ITCs did not change the level of HDAC3, and release to drug-free medium upregulated its level after 3 h of culturing, while it decreased at later times.

Next, we used another model of noncancerous cells, PNT2 normal adult prostatic epithelial cells, to analyze their response to ITCs. As shown in Fig. 5g, 3-h treatment with SFN or PEITC resulted in inhibition of DNA replication in PNT2 cells. It also moderately increased γH2A.X level, which lowered after further incubation in ITC-free medium (Fig. 5e). Much more pronounced DSB was induced by etoposide; however, it dropped efficiently already after 3-h culture in a drug-free medium, which resembles response of HDFa fibroblasts to DNA-damaging agents (Fig. 5b, f for comparison).

## Discussion

Genotoxic stress is one of the mechanisms underlying anticancer activity of ITCs. Induction of DNA damage checkpoints with the phosphorylation of H2A.X histone, activation of repair systems, and cell-cycle arrest have been reported for ITCs, including AITC and SFN [5, 9, 10, 19]. As γH2A.X histone is a marker of DSB, it was believed that ITCs induce this kind of DNA damage, which was also confirmed by comet assays [11] or constant field gel electrophoresis of DNA (CFGE) [10]. Results presented in this work also indicate that SFN and PEITC induce DSB in prostate cancer and to lesser extent—in normal cells. On the other hand, Sestili et al. reported that SFN induced single-strand breaks (SSB) in DNA of leukemia (Jurkat) and umbilical vein endothelial (HUVEC) cells [18]. Various cell models and treatment conditions (ITCs’ concentrations and incubation times) have been applied in these studies which might explain different results.

The question unresolved so far was about the mechanism of the DNA damage induced by ITCs. Numerous experimental data indicate that ITCs induce oxidative stress in cancer cells which contributes to their cytotoxicity [9, 12, 13, 18, 23–25]. Oxidative stress in ITC-treated cells results from inhibition of mitochondrial respiratory chain complexes and depletion of reduced glutathione [12, 13, 18, 23, 26]. It has been reported that SFN-induced SSB in DNA of Jurkat and HUVEC cells paralleled the kinetic of ROS elevation, and blocking ROS, especially of mitochondrial origin, protected against DNA damage [18]. On the other hand, only transient DNA oxidative modification (8-oxo-dG) has been reported in lung cancer cells treated with SFN for 0.5 h, but it was not detected at later timepoints when ROS production was significant [14]. Similarly, in prostate cancer cells, activation of DNA damage checkpoint kinases preceded peak of ROS formation [9, 12]. Strikingly, ascorbic acid or trolox did not attenuate PEITC-induced DNA damage in HCT116 colon cancer cells [11]. Thus, it seems that oxidative stress, even if contributes to ITCs cytotoxicity, might not be a key factor affecting DNA integrity in ITC-treated cells which is also in agreement with our results (Fig. 1c).

Another possibility is that ITCs inhibit enzymes engaged in DNA metabolism, which results in DNA breaks. In fact, it has been shown that higher level of DSB in Ras transformed cells compared with normal cells treated with BITC, PEITC or SFN depended on the level of topoisomerase 2α, which is a target for thiol modification by ITCs. ITCs induced the concentration-dependent reversible DNA cleavages in the presence of hTop2α in vitro [15].

Here, we show that SFN and PEITC inhibited DNA replication in both, cancer and noncancerous cells, which was evident already after 30 min of treatment. It did not depend on ROS of mitochondrial origin which we tested using Rho0 derivatives of PC-3 prostate cancer cells, that do not elevate ROS upon SFN [16]. Importantly, block in DNA replication preceded an increase in γH2A.X level that was significant in cancer cells after 2 h of exposition to ITCs. Thus, DSB induced by ITCs resulted from perturbed DNA replication rather than caused DNA replication block. Similar results have been shown for AITC which induced replication stress-associated DNA damage in human lung cancer cells, where FANCD2 repair protein formed foci at stalled or collapsed replication forks, ATM/ATR, Rad18, and Chk1 were activated, and γH2A.X level was elevated [5].

Inhibition of DNA replication might result from inhibition of Top2α [15] or other replication enzymes, such as proliferating cell nuclear antigen (PCNA) identified as potential SFN target protein [27]. It can also be caused by modification of chromatin by tested compounds. ITCs were shown to inhibit histone deacetylases (HDAC) activities [28] which led to elevated acetylation of histones H3 and H4 and activation of transcription of genes coding for negative cell-cycle regulators (p21) and pro-apoptotic proteins (Bax). Histone acetylation status is also important in DNA replication, both by regulating accessibility of origins to replication proteins during initiation phase and by facilitating removal of histones from DNA template and their reloading on daughter molecules during the elongation step (for review, see [29]). It has been reported that synthetic HDAC inhibitor, SAHA, slowed down replication forks and activated dormant origins which led to induction of DSB in DNA of breast cancer cells [21]. Here, we show that, indeed, ITCs inhibited HDAC I/II in tested cells; however, degree of this inhibition was comparable in PC-3 and HDFa cells. Thus, HDAC-inhibiting activity of ITCs might contribute to slowing down replication forks, but it does not explain varied levels of DSB in these cell lines. Although replication is blocked by ITCs in PC-3 and HDFa cells, DSB are significantly elevated only in cancer cells. One can speculate that normal cells do not experience DNA damage upon ITCs treatment which might be connected with their lower proliferation index comparing with cancer cells. To validate this hypothesis, we slowed down proliferation of PC-3 cells by culturing them in serum-deprived medium. Our results indicate that growth retardation slightly increased rather than reduced the level of γH2A.X. Thus, the growth rate is rather not responsible for lower DSB level in normal cells.

Replication stress should induce S-phase checkpoint, and indeed, SFN and PEITC increased the fraction of S-phase-arrested normal and cancer cells after 6 h of treatment (ca 8 or 11% in SFN- or PEITC-treated cells, respectively, vs 5% in control HDFa cells, and 16% in ITCs- treated cells vs 12% in control PC-3 cells). S-phase arrested HDFa cells were also observed after longer exposure to ITCs (16 h), but reached the control level after 24-h treatment (ca 8–9%). Interestingly, in the case of PC-3 cells, S-phase arrest was transient, and after longer exposure time, cells were able to progress to G2/M phase. Similarly, etoposide caused accumulation of PC-3 cells in both S and G2/M phases, with concomitant drop in the amount of G0/G1 cells, which was not observed in HDFa cells after 24-h treatment. It might indicate that in normal cells, the checkpoint machinery operates more efficiently that in cancer cells. It may also lead to more efficient DNA repair. To validate such possibility, we compared the levels of γH2A.X in cells treated for 3 h with ITCs and released to medium devoid of SFN or PEITC for additional 3 or 16 h. We observed that reduction of γH2A.X after ITCs removal was more efficient in normal cells, both fibroblasts (HDFa) and prostate epithelial cells (PNT2), than in PC-3 prostate cancer cells. It is much more evident in cells treated with etoposide which induces DSB to higher extent than ITCs.

More efficient DNA repair in normal cells was confirmed by immunoblotting for repair proteins. Human replication protein A (RPA) is a heterotrimeric complex of RPA70, RPA32, and RPA14 which binds single-stranded DNA and is necessary for DNA replication, repair, and recombination. In response to DNA damage, RPA is hyperphosphorylated and RPA32 phosphorylation occurs in at last nine positions, including Ser-4 and Ser-8. It is believed that it is catalyzed by PI3K-like kinases, including DNA-PK, ATM, and ATR (for review, see [30]). Both p-RPA32 and CtIP levels increased in ITC-treated HDFa and PC-3 cells (although in latter one to a higher degree). However, after ITCs removal, the level of these proteins dropped very rapidly in HDFa, while in PC-3 cells were maintained at constant level (CtIP- decreased after 16 h) which indicates that the damage has not been repaired efficiently in cancer cells. Contrary to our results, it has been demonstrated that CtIP level dropped in ITC-treated HCT116 colon cancer cells due to inhibition of activity and decrease in HDACs protein levels. Particularly, drop in HDAC3 led to an increased acetylation and degradation of CtIP [19]. Our results also indicate the HDAC activity inhibition; however, the level of HDAC3 is rather slightly elevated than lowered by ITCs which might explain elevated level of CtIP. Such discrepancies in the results may result from cell-type-specific effects or differences in experimental conditions.

It is well known that cancer cells often exhibit defects in the DNA repair mechanisms as well as checkpoint signaling which drives genomic instability characteristic for tumorigenesis process [31]. On the other hand, defects in DNA repair pathways can be exploited to potentiate DNA damage induced by anticancer drugs selectively in cancer cells [32, 33]. Documented selectivity of ITCs might rely on inherent features of healthy vs transformed cells concerning DNA repair. However, it is possible that ITCs, additionally to induction of DSB, inhibit some repair enzymes. Recently, it has been shown that SFN, at low concentrations, inhibited repair of DNA damage induced by (+)-anti-benzo[*a*]pyrene7,8-diol-9,10-epoxide in HCT116 colon cancer cells. It was connected with the inhibition of XPA, nucleotide excision repair enzyme, and SFN provoked zinc release from its zinc-binding domain [34]. Such domains are present in other proteins engaged in DNA repair, such as poly(ADP-ribose)polymerases or mammalian DNA ligase III [35]; thus, it is not excluded that ITCs modulate their activities as well.

In conclusion, our results indicate that ITCs block DNA replication in normal and cancer cells which precedes DSB. However, DNA damage is more potent in cancer cells due to less efficient DNA repair compared with noncancerous cells (Fig. 5h). Novelty of this work relies on analysis of ITCs impact on DNA metabolism (replication and damage and repair) in cancer and noncancerous cells. Obtained results might explain, at least partially, higher antiproliferative activity of ITCs against cancer cells and provide the starting point for research on therapies combining ITCs and drugs damaging DNA or blocking its replication.
